# Metabolic, genetic and immunological features of relatives of type 1 diabetes patients with elevated insulin resistance

**DOI:** 10.1007/s40618-024-02497-x

**Published:** 2024-12-10

**Authors:** V. Codazzi, V. Salvatore, F. Ragogna, I. Marzinotto, A. Anselmo, N. Baldoni, M. R. Pastore, S. Martinenghi, A. Stabilini, E. Bosi, A. Giustina, L. Piemonti, I. Libman, H. M. Ismail, M. J. Redondo, V. Lampasona, P. Monti, A. Giovenzana, A. Petrelli

**Affiliations:** 1 IRCCS Ospedale San Raffaele, Via Olgettina 60, Milan, 20132 Italy; 2 UPMC Children’s Hospital of Pittsburgh, Pittsburgh, USA; 3 Indiana University School of Medicine, Indianapolis, USA; 4 Texas Children’s Hospital, Baylor College of Medicine, Houston, USA; 5 Present Address: University of Milan and Pio Albergo Trivulzio, Milan, Italy

**Keywords:** Insulin resistance, Type 1 diabetes, T cells, T1D genetic risk score, Metabolic hormones

## Abstract

**Purpose:**

Insulin resistance plays a pivotal role in the preclinical stages of type 1 diabetes (T1D).

**Objective:**

This study aims at exploring the genetic, metabolic, and immunological features associated with insulin resistance among individuals at risk of developing T1D.

**Methods:**

We retrospectively selected relatives of individuals with T1D from participants in the TrialNet Pathway to Prevention study. They were categorized into two groups: high-H (n = 27) and low-H (n = 30), based on the upper and lower quartiles of insulin resistance assessed using the Homeostatic Model Assessment of Insulin Resistance (HOMA-IR). Genetic predisposition was determined using the T1D Genetic Risk Score 1 (GRS1). Additionally, glucose control was evaluated through an oral glucose tolerance test and levels of metabolic hormones and inflammatory cytokines were measured in the serum. Flow cytometry analysis was employed to assess frequency and phenotype of islet-specific CD8 T cells.

**Results:**

While GRS1 were similar between the low-H and high-H groups, high-H individuals displayed a distinct metabolic profile, characterized by compensatory hyperinsulinemia, even while maintaining normoglycemia. Circulating cytokine levels were similar between the two groups. However, immune profiling revealed a central memory and activated profile of GAD65-specific CD8 T cells, along with an increased frequency of insulin-specific CD8 T cells in high-H individuals. The enrichment in insulin-specific CD8 T cells was independent of body mass.

**Conclusion:**

These findings highlight the intricate interplay between insulin resistance, genetic factors, and immune activation in the context of T1D susceptibility, indicating potential connections between insulin resistance and immune responses specific to islet cells.

**Supplementary Information:**

The online version contains supplementary material available at 10.1007/s40618-024-02497-x.

## Introduction

Insulin resistance, often linked to excess body weight, is characterized by a diminished responsiveness of insulin-sensitive tissues to insulin action. This results in an increase in compensatory insulin production, ultimately leading to the impairment of β-cell function, which is the underlying mechanism for the development of type 2 diabetes (T2D) [1]. In contrast, type 1 diabetes (T1D) arises from an autoimmune assault on pancreatic β-cells, where islet autoantibodies can be detected in serum years before clinical T1D onset, enabling the identification of individuals at risk [2, 3].

While T1D and T2D have historically been perceived as distinct, emerging evidence suggests shared pathogenic components [4]. Indeed, an increasing number of T1D patients exhibit a perturbed metabolic profile characterized by obesity and/or insulin resistance [5, 6]. Notably, insulin resistance plays a pivotal role even prior to clinical T1D onset, with several studies demonstrating that autoantibody-positive relatives of individuals with T1D, acknowledged to be at high risk of developing the disease [7], face an even increased risk of T1D development when accompanied by increased insulin resistance and/or excess body weight [8–12]. These findings suggest that insulin resistance may function as an accelerator for disease onset, as postulated by the accelerator hypothesis [13]. However, a consensus regarding the mechanisms through which insulin resistance may expedite the onset of T1D and the precise relationship between insulin resistance and autoimmunity remains elusive.

While several studies have independently investigated the genetic, metabolic, and immunological characteristics of relatives of T1D patients [14–20], the impact of insulin resistance on these traits remains unexplored. Therefore, the objective of our study is to characterize the genetic, metabolic, and immunological attributes associated with insulin resistance in a cohort of subjects with elevated susceptibility to the development of T1D, such as relatives of T1D patients.

## Materials and methods

### Subjects enrolled in the study

Participants of this study were selected retrospectively from a cohort of first- and second-degree relatives of individuals with type 1 diabetes enrolled in the Type 1 Diabetes TrialNet Pathway to Prevention Trial (TNPTP) [21] at the TrialNet Clinical Center of the IRCCS Ospedale San Raffaele (OSR) from June 2012 to July 2019. TNPTP participants were screened for islet autoantibodies to glutamic acid decarboxylase (GADA), insulin (microinsulin antibody assay, mIAA), insulinoma-associated antigen 2 (IA-2A), zinc transporter 8 (ZnT8A) and islet cell antibodies (ICA). TNPTP participants who tested positive for autoantibodies in at least one of their previous visits were monitored with autoantibody testing, glycated hemoglobin (HbA1c), and a 2-h oral glucose tolerance test (OGTT) at 6-month intervals. Stage 1 was defined as the presence of ≥ 2 autoantibodies and normoglycemia (fasting glucose < 5.6 mmol/l and/or 2-h glucose < 7.8 mmol/l). Stage 2 was defined as the presence of ≥ 2 autoantibodies and dysglycemia (fasting glucose ≥ 5.6 mmol/l and/or 2-h glucose ≥ 7.8 mmol/l), while Stage 3 was defined as diabetes (fasting glucose ≥ 7.0 mmol/l and/or 2-h glucose ≥ 11.1 mmol/l). The complete TNPTP protocol is available online (http://www.clinicaltrials.gov/show/NCT00097292). The TNPTP study was approved by the OSR Ethics Committee (IRB# NHPROT32803-TN01). This project was performed using additional blood samples drawn during the visits for ancillary studies (IRB#TIGET004-DRI003).

Out of the 1316 relatives enrolled in the TNPTP cohort at OSR (OSR-TNPTP), 142 individuals were autoantibody-positive and underwent OGTT as per the TNPTP protocol. Inclusion criteria required the availability of glucose, insulin and C-peptide levels during a 2-h OGTT. Exclusion criteria comprised: (1) clinical manifestations of infection, such as fever, cough, or cold, within the two weeks preceding the study visit during which the OGTT was performed; and (2) concurrent treatments, encompassing antibiotics, immunosuppressive agents, or insulin therapy. Following these selection criteria, a total of 125 individuals were retrospectively chosen for inclusion in the current study. Each participant contributed data only once.

**Fig. 1:**
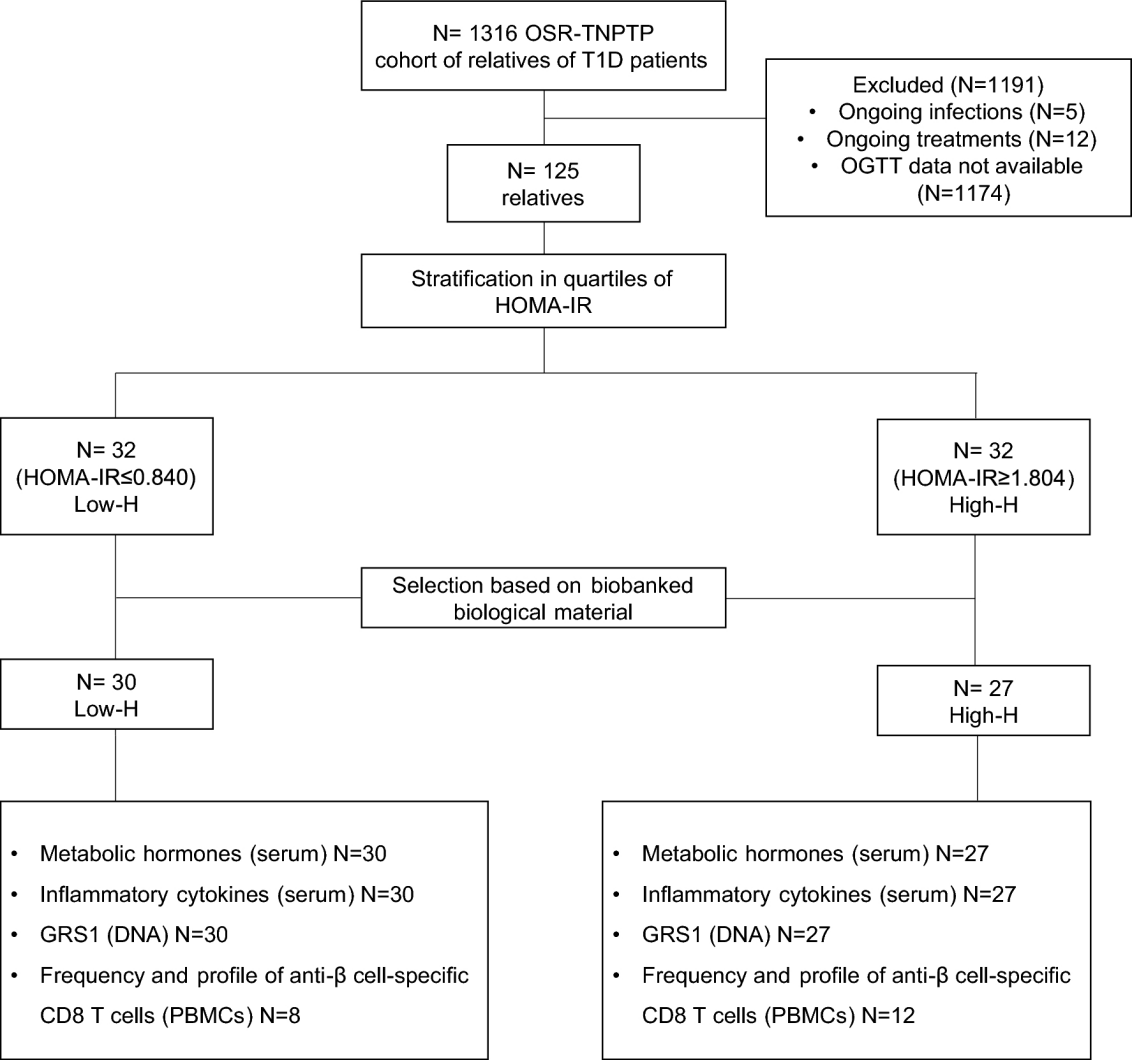
Study design. 125 subjects were selected starting from a cohort of 1316 relatives of individuals with T1D, enrolled in the TrialNet Pathway to Prevention Trial (TNPTP) at the IRCCS Ospedale San Raffaele (OSR), based on the inclusion and exclusion criteria, including the availability of data from 2-h oral glucose tolerance test (OGTT). The Homeostatic Model Assessment of Insulin Resistance (HOMA-IR) was utilized as an indicator of insulin resistance to categorize relatives into quartiles. The lower and upper quartiles were identified, resulting in groups of 32 relatives with low insulin resistance (low-H) and 32 relatives with high insulin resistance (high-H), respectively. The groups were further reduced to 30 relatives for the low-H group and 27 for the high-H group based on the availability of biological materials. Various assays were conducted on this selected cohort, including the analysis of metabolic hormones and inflammatory cytokines in serum, the assessment of the T1D genetic risk score 1 (GRS1) using DNA, and the characterization of β-cell-specific CD8 T cells in peripheral blood mononuclear cells (PBMCs)

Selected individuals were then categorized into quartiles of insulin resistance, determined by Homeostatic Model Assessment of Insulin Resistance (HOMA-IR), with both the upper and lower quartiles included in the study. By specifically focusing on patients at the extremes of insulin resistance, our objective was to increase the probability of identifying potential metabolic, genetic and immunological disparities. Subsequently, an additional selection process was carried out based on the availability of biobanked biological samples (i.e., serum, whole blood, and peripheral blood mononuclear cells), collected during the same visit as the OGTT. The final cohort of chosen relatives consisted of n = 30 individuals for the low insulin resistance group (low-H) and n = 27 for the high insulin resistance group (high-H) (refer to Fig. 1 for the study design).

### 2-h Oral Glucose Tolerance Test (OGTT)

Autoantibody-positive participants underwent an OGTT (oral glucose load dose 1.75 g/kg, maximum 75 g) after an overnight fast. C-peptide (ng/ml), glucose (mmol/l) and insulin (pmol/l) measurements were performed fasting and then after oral glucose intake at 30, 60, 90, and 120 min.

### Metabolic indices

For the metabolic characterization of our cohort, indices of insulin sensitivity and beta cell function were employed. Insulin resistance was calculated using the HOMA-IR [22]. The Matsuda index, which measures insulin sensitivity, is expressed by the ratio of 10,000 to the square root of the product between fasting glucose, fasting insulin, mean glucose and mean insulin during OGTTs [23]. Dysglycemia was defined as fasting glucose ≥ 5.6 mmol/l and/or 2-h glucose ≥ 7.8 mmol/l. The C-peptide area under the curve (AUC) was calculated using the trapezoidal rule, while the Insulinogenic Index (IGI) as the ratio of insulin (pmol/l) at 30 min minus fasting insulin (pmol/l) to glucose (mmol/l) at 30 min minus fasting glucose (mmol/l). Both are strong predictors of β-cell function [24, 25].

### BMI percentile (BMIp) calculation

Body mass index percentiles (BMIp) were determined based on sex- and age-specific charts from the Italian population (Italian.ref) of the childsds R package using R statistical Software (version 4.3.0).

### Proinsulin/C-peptide ratio

The ratio between proinsulin (PI) and C-peptide has been used as a marker of endoplasmic reticulum stress in β-cells and predictor for β-cell dysfunction [26]. Quantification of proinsulin in the serum was determined by enzyme-linked immunosorbent assay (ELISA) (Human Total Proinsulin Elisa kit, EMD Millipore, Merck; cat# EZHPI-15 K, RRID: AB_3099413) and BioTek Epoch Microplate Spectrophotometer. C-peptide was measured with the Hormone Magnetic Bead Panel (EMD Millipore, Merck; cat # HMHEMAG-34 K) and Bio-plex MAGPIX Multiplex Reader (BIO-RAD).

### Serum metabolic hormones and adipokines

The gastric inhibitory polypeptide (GIP), leptin, pancreatic polypeptide (PP), active amylin and insulin (I) were measured in serum samples withdrawn after an overnight fasting using the Human Metabolic Hormone Magnetic Bead Panel (EMD Millipore, Merck; cat # HMHEMAG-34 K). Adiponectin, adipsin, resistin and plasminogen activator inhibitor-1 (PAI-1) were measured in the serum of fasting subjects with the Human Adipokine Magnetic Bead Panel (Millipore, Merck; cat # HADK1MAG-61 K). Measures were performed using the Bio-plex MAGPIX Multiplex Reader (BIO-RAD). The Bio-Plex Manager MP Software was used for data acquisition, quantification, and optimization of standard curves for metabolic hormones and adipokines; final concentrations are expressed in pg/mL.

### DNA extraction and T1D genetic risk score 1

DNA was extracted from whole blood samples using the Maxwell® RSC Instrument and the Maxwell® RSC Blood DNA Kit by Promega (cat #AS1400). The T1D genetic risk score 1 (GRS1), based on 30 SNPs associated with an increased risk of T1D [15], was calculated for each subject by summing the contribution of each allele as the product of the dosage and the natural logarithm of the odd ratio [27]. Four SNPs associated with the HLA class II risk haplotypes DR3 and DR4-DQ8 were analysed by droplet digital PCR (Bio-Rad, USA). All other risk SNPs were typed using the high-throughput OpenArray system (Life Technologies, USA) [28].

### Inflammatory cytokines

Growth related oncogene-alpha (GRO α), interferon-alpha2 (IFNα2), interferon gamma (IFNγ), interleukin 1 beta (IL-1β), interleukin 1 receptor antagonist (IL-1RA), interleukin 13 (IL-13), interleukin 15 (IL-15), interleukin 17-A (IL-17A), interleukin 6 (IL-6), interleukin 8 (IL-8), interleukin 10 (IL-10), interferon-gamma inducible protein 10 kDa (IP 10), monocyte chemoattractant protein 1 (MCP1), macrophage inflammatory protein-a alpha (MIP-1α), soluble CD40 ligand (sCD40L) and tumor necrosis factor alpha (TNFα) were measured in the serum samples using the Human Cytokine/Chemokine/Growth Factor Panel A Magnetic Bead Panel (EMD Millipore, Merck; cat# HCYTA-60 K) and the Bio-plex MAGPIX Multiplex Reader -BIO-RAD. Final concentrations are expressed in pg/mL.

### PBMCs isolation

After blood withdrawal, peripheral blood mononuclear cells (PBMCs) were isolated by density gradient centrifugation using Lympholyte® Cell Separation density gradient centrifugation media (Cedarlane, cat # CL5020) and frozen in Roswell Park Memorial Institute (RPMI) supplemented with dimethyl sulfoxide (DMSO) (10%) and fetal bovine serum (20%) for further experimentation.

### Flow cytometry analysis

From the cohort of 57 relatives selected for this study, 20 subjects—who tested positive for HLA-A2 (using PE anti-human HLA-A2 antibody, Biolegend, cat #343306, RRID: AB_1877228)—were selected to perform flow cytometry analysis for the study of islet-specific CD8 T cells. PBMCs were thawed and resuspended in fresh RPMI + 10% FBS (RPMI + 10% FBS + 1% glutamine + 1% P/S). First, cells were washed and stained for dead cell exclusion using BD Horizon™ Fixable Viability Stain 575 V (RRID: AB_2869702) at RT for 15 min in phosphate-buffered saline (PBS). Then, samples were washed and stained with insulin and GAD65-specific dextramers at RT for 10 min in FACS buffer (PBS + 1% bovine serum albumin + 0.1% sodium azide). Finally, cells were incubated using appropriate saturating concentrations of the following conjugated monoclonal antibodies; CD45 (RRID: AB_1236444), CD3 (RRID: AB_2744382), CD56 (RRID: AB_2916880), CD4 (RRID: AB_2870176), CD8 (RRID: AB_1645481), CD45RA (RRID: AB_2660984), CCR7 (RRID: AB_2033948), CD69 (RRID: AB_2561783), CD154 (RRID: AB_2743658), CD137 (RRID: AB_2871191) and LAG3 (RRID: AB_2869706), at RT for 20 min in FACS buffer. The list of anti-human monoclonal antibodies and anti-GAD65 and anti-insulin dextramers, including company, catalogue number, clone, peptide sequence (for dextramers) and concentration used, is provided in Supplementary Table 1 [29]. The FACSymphony™ A5 analyzer (BD Bioscience) was used for sample acquisition. Instrument performance was checked daily using CS&T Beads (BD Bioscience) and SPHERO Rainbow Beads (Spherotech Inc., Lake Forest, IL). Data acquisition and analyses were performed using FACSDiva v.8 (BD Pharmingen), and Flow-jo v.10 (Tree Star Inc.) respectively.

### Statistical analysis

To ensure the representativeness of the population for genetic, metabolic, and immunological analyses, quartiles of the HOMA-IR index were calculated on the final OSR-TNPTP cohort consisting of 125 relatives. This decision was made as the International TNPTP cohort demonstrated significantly higher levels of HOMA-IR (OSR-TNPTP: mean 1.42, SD ± 0.80 vs. International TNPTP: mean 1.75, SD ± 1.57, p < 0.0001). The cohort of relatives of individuals with T1D was stratified based on quartiles, with Q1 = 0.840, Q2 or median = 1.320 and Q3 = 1.804. Subjects falling in the lower and upper quartiles were identified as the low insulin resistance group (low-H, HOMA-IR ≤ 0.840) and the high insulin resistance group (high-H, HOMA-IR ≥ 1.804), respectively. Data are summarised in tables as median and interquartile range (IQR) or as absolute number and percentage. Graphs are presented as median and interquartile range (IQR). Statistical analyses were performed with GraphPad Prism V.9.00 software (GraphPad). When comparing continuous variables of two independent groups, data were analysed using the Mann–Whitney test. In the case of comparison between multiple groups the Kruskal–Wallis test and Dunn’s multiple comparison test were used. Fisher’s exact test was used for comparison between nominal variables. To test the correlation among continuous parameters, the Spearman’s rank-order correlation analysis was performed. To determine the effect of BMIp on relationships between variables of interest and HOMA-IR, two linear models were constructed using R (version 4.3). In the first model, each variable of interest was modeled solely as a function of the HOMA-IR group variable. In the second model, each variable was considered a dependent variable, with BMIp and HOMA-IR group as independent variables. Missing BMIp values were removed prior to analysis to avoid bias. Data are considered statistically different when p value was < 0.05; *graphically indicates p < 0.05, **graphically indicates p < 0.01, ***graphically indicates p < 0.001, ****graphically indicates p < 0.0001.

## Results

### Metabolic and hormonal profiles of high-H relatives

**Fig. 2:**
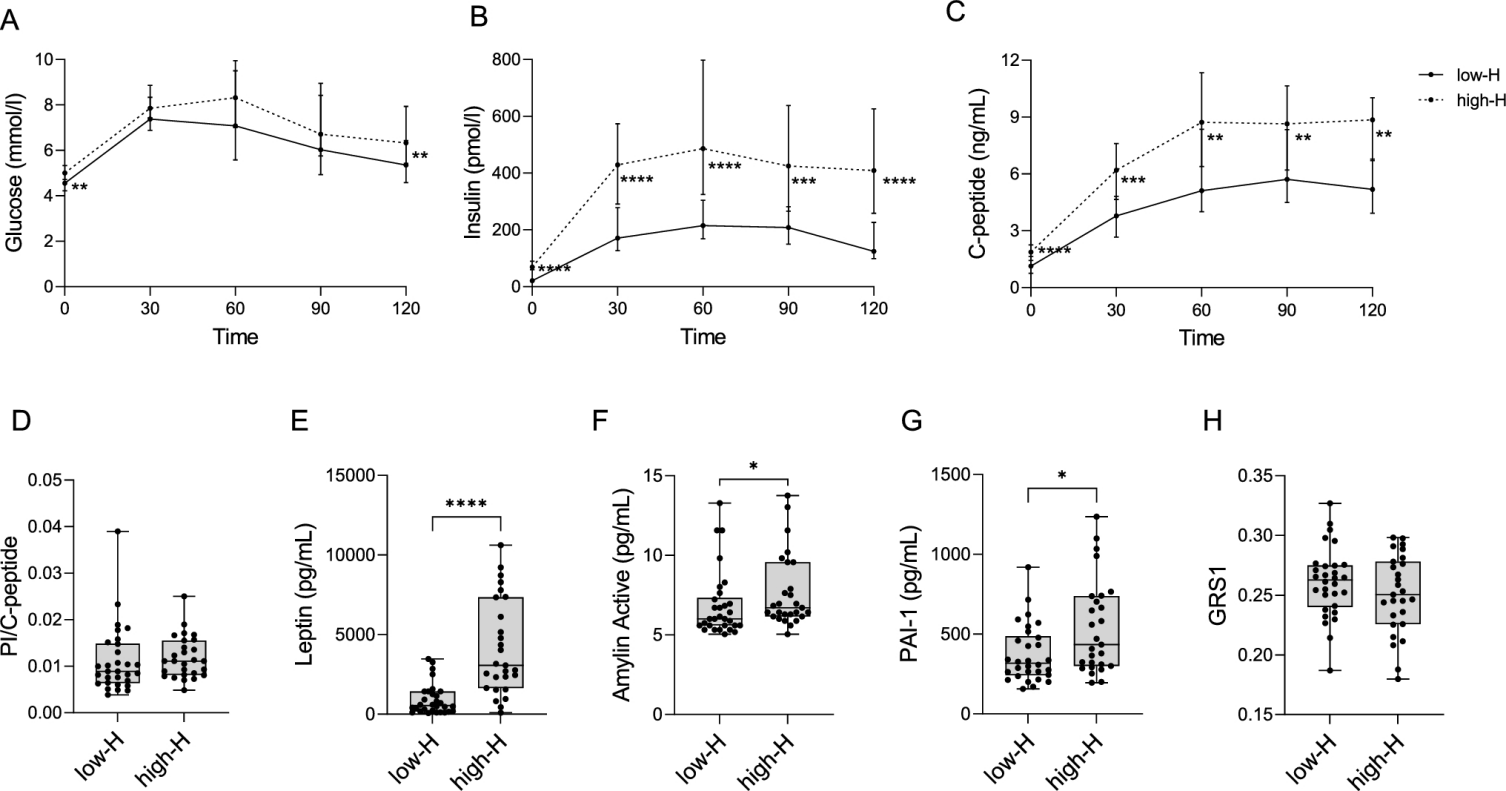
Metabolic Profile and Genetic Risk of high-H vs. low-H relatives of T1D patients. Curves illustrating glucose (mmol/l) (**A**), insulin (pmol/l) (**B**), and C-peptide (ng/mL) (**C**) levels during various time points (0, 30, 60, 90, and 120 min) of the OGTT are shown. The continuous line represents the low-H group, while dashed line represents the high-H group. Comparison of the Proinsulin (PI) to C-peptide ratio between low-H and high-H groups (**D**). Comparison of leptin levels between low-H and high-H groups (**E**). Comparison of active amylin levels between low-H and high-H groups (**F**). Comparison of plasminogen activator inhibitor-1 (PAI-1) levels between low-H and high-H groups (**G**). Comparison of T1D Genetic Risk Score 1 (GRS1) between low-H and high-H groups (**H**). All graphs are presented as the median and interquartile range (IQR). Statistical significance was determined using the Mann–Whitney test. *p < 0.05, **p < 0.01, ***p < 0.001, ****p < 0.0001. *PI* Proinsulin*; PAI-1* Plasminogen Activator Inhibitor-1*; GRS1* T1D Genetic Risk Score 1

A total of n = 125 subjects was selected from our local TNPTP cohort (OSR-TNPTP) of relatives of T1D patients; selected samples were representative of the whole OSR-TNPTP cohort of 1316 relatives (sex: p = 0.35; age: p = 0.28). Relatives of T1D patients with available OGTT data were stratified based on their HOMA-IR levels, with subjects in the upper quartile categorized as the high-H group and those in the lower quartile as the low-H group. The two groups were homogenous for sex, age and number of autoantibodies (Table 1). Differences emerged in the metabolic profiles with high-H relatives displaying higher BMIp and reduced Matsuda index as well as enhanced β-cell function, the latter indicated by elevated values for IGI and C-peptide AUC (Table 1). While the levels of HbA1c, the prevalence of dysglycemia and the number of subjects in Stages 1 or 2 were comparable between groups (Table 1), high-H individuals exhibited higher fasting glucose levels as well as elevated glucose levels 2-h following oral glucose consumption (Fig. 2A). This was accompanied by increased insulin production (Fig. 2B) and consistently higher C-peptide levels at all time points during the OGTT (Fig. 2C). Importantly, no signs of β-cell stress, as measured by the PI/C-peptide ratio, were observed in comparison to low-H subjects (Fig. 2D). Moreover, high-H relatives showed increased levels of obesity-related metabolic hormones, including leptin, PAI-1, and active amylin (Fig. 2E-G), both positively correlated with HOMA-IR (Supplementary Fig. 1A-C, with only a trend observed for PAI-1) [29]. However, no significant differences were observed in the other metabolic hormones examined (Supplementary Fig. 2) [29].

**Table 1:** Clinical characteristics of low-H and high-H relatives of T1D patients

	Low-H (n=30)	High-H (n=27)	p value
Sex, F (%)	14 (46.7)	17 (63.0)	p = 0.29
*Age (yrs)*	19.2 (9.1-34.5)	14.0 (10.7-19.2)	p = 0.38
<18 yrs	8.7 (5.5-11.9) n=14	11.6 (9.2-14.5) n=19	p = 0.06
>18 yrs	32.8 (26.1-37.7) n=16	20.1 (19.4-45.4) n=8	p = 0.46
BMIp	37.3 (15.6-51.4) n=27	53.6 (28.6-91.9) n=27	p = 0.006
HOMA-IR	0.6 (0.5-0.7)	2.4 (1.9-2.8)	p < 0.0001
Matsuda	12.5 (9.8-14.3) n=30	3.8 (3.4-5.1) n=26	p < 0.0001
IGI	59.0 (29.3-80.7) n=30	150.6 (79.4-162.9) n=26	p = 0.0003
C-peptide (AUC)	568.0 (387.2-843.4) n=30	856.9 (707.7-1081.0) n=26	p = 0.0004
HbA1c %	5.0 (4.8-5.1) n=28	5.1 (4.8-5.4) n=27	p = 0.23
Dysglycemia, n (%)	7 (23.3)	8 (29.6)	p = 0.76
*N Aab*			
0, n (%)	4 (13.3)	3 (11.1)	
1, n (%)	14 (46.7)	14 (51.9)	
2, n (%)	4 (13.3)	4 (14.8)	
3, n (%)	5 (16.7)	4 (14.8)	
4, n (%)	1 (3.3)	2 (7.4)	
5, n (%)			
Unknown, n (%)	2 (6.7)		
*Type of Aab*			
GAD65, n (%)	16 (53.3)	23 (85.2)	
ZnT8, n (%)	5 (16.7)	7 (25.9)	
IA2, n (%)	4 (13.3)	4 (14.8)	
mIAA, n (%)	7 (23.3)	3 (11.1)	
ICA, n (%)	9 (30.0)	5 (18.5)	
Stage 1 T1D	7 (23.3)	6 (22.2)	
Stage 2 T1D	3 (10.0)	4 (14.8)	

Data are expressed as the median and interquartile range for continuous variables (age, BMIp, HOMA-IR, Matsuda, IGI, C-peptide (AUC), HbA1c), and as absolute numbers and percentages for nominal variables (sex, dysglycemia, number and type of autoantibodies positive patients, number of patients in Stages 1 and 2 T1D). P-values were calculated using the Mann-Whitney test for continuous variables and Fisher’s exact test for nominal variables. *BMIp *Body Mass Index Percentile; *HOMA-IR* Homeostatic Model Assessment of Insulin Resistance;* IGI *Insulinogenic Index;* AUC *Area Under the Curve; *HbA1c *Glycated Hemoglobin

In summary, individuals with high-H demonstrated initial metabolic alterations, characterized by compensatory hyperinsulinemia, while maintaining overall normoglycemia.

### Genetic predisposition to T1D in high-H relatives

To assess whether the genetic predisposition to T1D varies between low-H and high-H individuals, we calculated the GRS1 in our cohort. GRS1, comprising a weighted sum of 30 SNPs associated to T1D risk, demonstrated no significant differences between the two groups (Fig. 2H), suggesting a comparable unmodifiable genetic predisposition to T1D. Furthermore, no correlations were observed between GRS1 and HOMA-IR (Supplementary Fig. 3) [29]. No specific SNP exhibited association with high-H individuals (data not shown).

Collectively, these findings suggest that the observed differences in insulin resistance between the low-H and high-H groups are not primarily driven by genetic predisposition to T1D.

### Frequency and phenotype of islet-specific CD8 T cell responses in high-H relatives

We investigated potential differences related to non-specific inflammation as well as islet-specific responses between individuals with low-H and high-H. Circulating levels of inflammatory cytokines showed no significant differences between the two groups (Supplementary Table 2) [29].

A subgroup of n = 20 HLA-A2^+^ individuals, comprising n = 8 low-H and n = 12 high-H participants, was selected from the primary cohort to determine the frequency of circulating islet-specific CD8 T cells by flow cytometry using dextramers. The newly-selected subgroups were representative of the whole low-H (sex: p > 0.99; age: p = 0.98; BMIp: p = 0.60) and high-H (sex: p > 0.99; age: p = 0.08; BMIp: p = 0.48) groups (characteristics of these individuals are available in Supplementary Table 3) [29]. The gating strategy used for the data analysis is shown in Supplementary Fig. 4 [29]. The frequency of GAD65-specific and insulin-specific cells on CD8 T cells was examined, with gates on CD4 T cells serving as negative controls (Supplementary Fig. 5) [29].

**Fig. 3:**
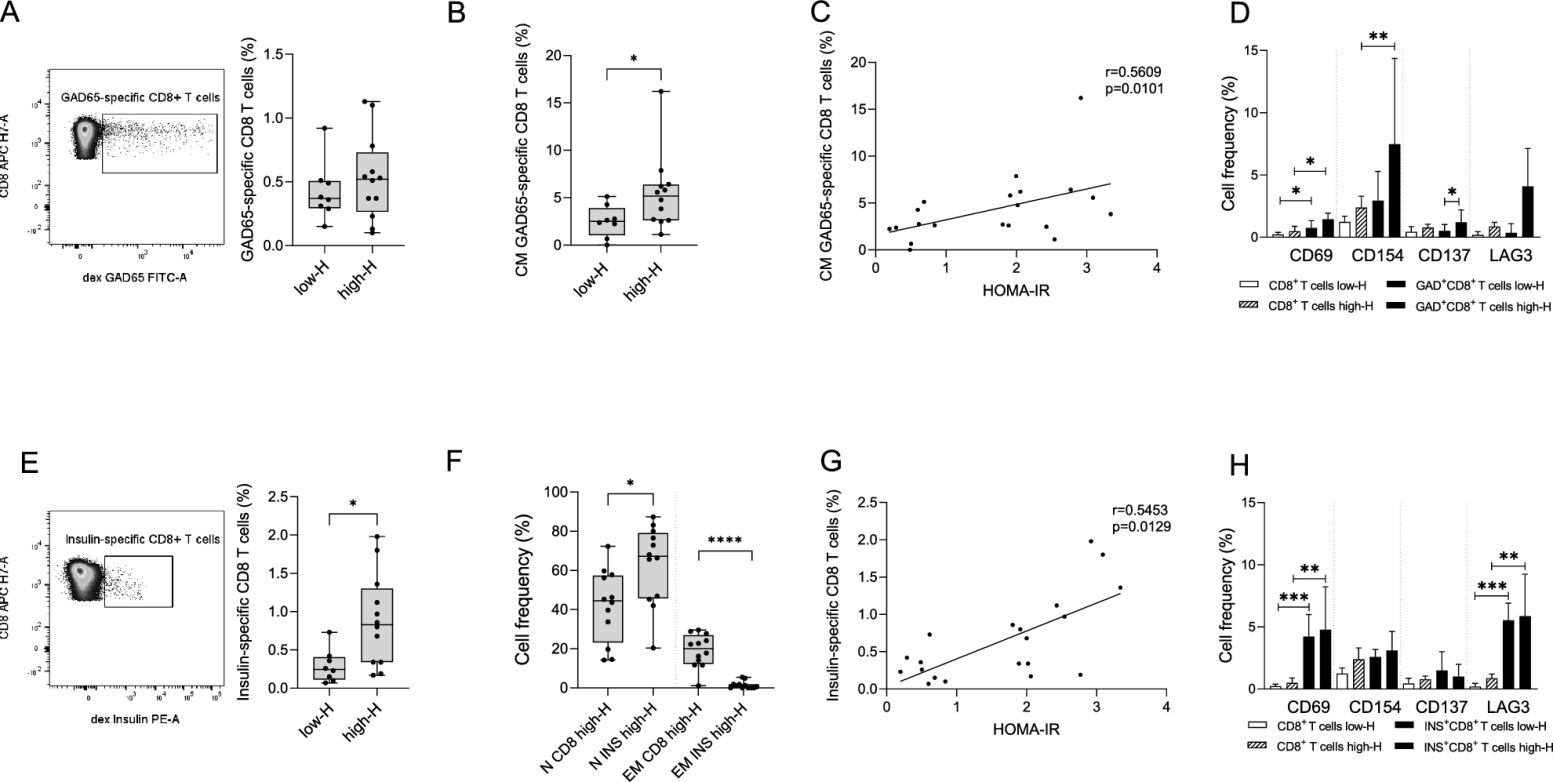
Islet-Specific CD8 T Cells in high-H vs. low-H HLA-A2 positive relatives of T1D patients. Representative dot plots showing GAD65-specific T cells within the CD8 T cell population (**A**, left panel) and comparison of GAD65-specific CD8 T cell frequency between low-H (n = 8) and high-H groups (n = 12) (**A**, right panel). Comparison of CM GAD65-specific CD8 T cell frequency between low-H and high-H groups (**B**). Correlation between CM GAD65-specific CD8 T cells and HOMA-IR (**C**). Comparison of CD69 +, CD154 +, CD137 +, and LAG3 + cell frequency between cell subsets (CD8 T cells and GAD-specific CD8 T cells) and between metabolic conditions (low-H and high-H) (**D**). Representative dot plots showing insulin-specific T cells within the CD8 T cell population (**E**, left panel) and comparison of insulin-specific CD8 T cell frequency between low-H and high-H groups (**E**, right panel). Comparison of N and EM T cells frequency between cell subsets (bulk CD8 T cells and insulin-specific CD8 T cells) of high-H group (**F**). Correlation between insulin-specific CD8 T cells and HOMA-IR (**G**). Comparison of CD69 +, CD154 +, CD137 +, and LAG3 + cell frequency between cell subsets (CD8 T cells and insulin-specific CD8 T cells) and between metabolic conditions (low-H and high-H) (**H**). All graphs are presented as the median and interquartile range (IQR). Statistical significance was calculated using the Mann–Whitney test for A, B, E, F, and the Kruskal–Wallis test with Dunn’s multiple comparison test for D and H. *p < 0.05, **p < 0.01, ***p < 0.001, ****p < 0.0001. Correlations were determined through the Spearman’s rank-order correlation analysis. *N* Naïve*; CM* Central Memory*; EM* Effector Memory*; HOMA-IR* Homeostatic Model Assessment of Insulin Resistance

The frequency of GAD65-specific CD8 T cells showed no significant difference between individuals with low-H and high-H (Fig. 3A) and exhibited a trend of correlation with HOMA-IR (r = 0.42, p = 0.05) (Supplementary Fig. 6A) [29]. High-H individuals exhibited an enrichment of GAD65-specific CD8 T cells with a central memory (CM) phenotype compared to low-H participants (Fig. 3B), with no observed differences in the frequency of naïve, effector memory (EM), and terminally differentiated (TEMRA) GAD65-specific CD8 T cells (data not shown). This enrichment positively correlated with HOMA-IR (r = 0.56, p = 0.01) (Fig. 3C) and Matsuda index (r = -0.54, p = 0.01) (Supplementary Fig. 6B) [29], suggesting a specific link between insulin resistance and anti-GAD65-specific CD8 T cells with a memory phenotype. Additionally, GAD65-specific CD8 T cells in high-H individuals exhibited higher activation compared to low-H, as evidenced by increased expression of CD137. CD69 and CD154 markers were also found to be elevated in GAD65-specific CD8 T cells when compared to bulk CD8 T cells (Fig. 3D).

The frequency of insulin-specific CD8 T cells was elevated in high-H relatives compared to their low-H counterparts (Fig. 3E). Furthermore, when compared to the bulk CD8 T cells compartment, these cells exhibited a predominantly naïve phenotype in contrast to the effector memory (EM) phenotype observed in bulk CD8 T cells of high-H subjects (Fig. 3F). No differences in the differentiation phenotype of insulin-specific CD8 T cells between low-H and high-H individuals were observed (Supplementary Fig. 7) [29]. Notably, the frequency of insulin-specific CD8 T cells positively correlated with HOMA-IR (r = 0.54, p = 0.01) (Fig. 3G) and Matsuda index (r = -0.56, p = 0.009) (Supplementary Fig. 8) [29], suggesting a link between insulin resistance and insulin-specific CD8 T cells. Furthermore, insulin-specific CD8 T cells from high-H exhibited increased expression of the activation markers CD69 and LAG3 compared to bulk CD8 T cells, but not to low-H individuals, suggesting that their activation is independent from insulin resistance (Fig. 3H).

Upon restratification of the cohort based on the Matsuda Index (n = 9 low-Matsuda vs. n = 8 high-Matsuda), our data confirmed an increase in CM GAD-specific CD8 T cells (p = 0.02) alongside elevated insulin-specific CD8 T cells (p = 0.04) in low-Matsuda individuals, both showing an inverse correlation with the Matsuda index (r = -0.53 and p = 0.03 for CM GAD-specific CD8 T cells; r = -0.57 and p = 0.02 for insulin-specific CD8 T cells).

To explore potential links between cell-mediated and humoral responses of the adaptive immune system, we correlated the frequency of GAD65-specific and insulin-specific CD8 T cells with anti-glutamic acid decarboxylase (GADA) and anti-insulin (mIAA) antibody levels, respectively. GAD65-specific CD8 T cells displayed a weak correlation with GADA levels (r = 0.44, p = 0.05) (Supplementary Fig. 9A) [29], while no significant correlation was observed between mIAA levels and insulin-specific CD8 T cells (r = 0.22, p = 0.35) (Supplementary Fig. 9B) [29].

In conclusion, our data reveal that high-H relatives of T1D patients are characterized by alterations in the frequency and phenotype of islet-specific CD8 T cells.

### Influence of BMIp on metabolic, genetic and immunologic characteristics of high-H relatives

To evaluate the impact of body weight on metabolic, genetic, and immunological parameters, we utilized a linear regression model with and without BMIp adjustment. Supplementary Table 4 [29] provides a comparison of unadjusted and BMIp-adjusted p-values. Overall, metabolic features in high-H individuals remained largely consistent regardless of BMIp adjustment, except for PAI-1, which showed sensitivity to obesity levels, and amylin active, where differences between high-H and low-H were inconclusive in unadjusted analyses. Genetic predisposition was unaffected by BMIp. A trend in CM GAD65-specific CD8 T cells was observed between low-H and high-H, persisting even in the unadjusted model. Importantly, the heightened expression of insulin-specific CD8 T cells in the high-H group was independent of BMIp, suggesting a specific relationship with insulin resistance. Additionally, GAD65-specific CD8 T cells exhibited increased expression of the LAG-3 activation marker in high-H individuals, unaffected by BMIp, unlike CD137. In summary, our findings suggest that BMIp minimally influences the metabolic profile of high-H individuals and does not impact genetic predisposition or the increased frequency of insulin-specific CD8 T cells.

## Discussion

Our findings demonstrate that high levels of insulin resistance among relatives of T1D patients are associated with distinctive metabolic and immunological profiles. Notably, the genetic factors conventionally linked to T1D susceptibility do not exert a prominent influence on insulin resistance.

In line with their insulin-resistant condition, our cohort of high-H relatives displayed distinctive metabolic profiles characterized by increased insulin secretion while maintaining normoglycemia. This is likely a consequence of physiological changes induced by insulin resistance, including reduced peripheral glucose uptake and elevated insulin demand [12]. Indeed, high-H relatives exhibited compensatory hyperinsulinemia without signs of β-cell stress or hyperglycemia, indicating enhanced β-cell functionality, required to sustain normoglycemia. These findings align with the 'accelerator hypothesis' postulated by Wilkin T.J., suggesting that insulin resistance leads to the metabolic overloading of β-cells and subsequent hyperglycemia. Both factors contribute to β-cell stress and apoptosis, ultimately facilitating autoimmunity [12, 13, 30]. Several studies have reported that high glucose concentrations play a pivotal role in triggering damage and autoimmunity in pancreatic islets [31, 32], with intermittent high glucose exposure potentially exacerbating β-cell toxicity [33]. This phenomenon is likely occurring in our high-H group.

Sustained hyperinsulinemia and elevated leptin levels, observed in the high-H group of relatives, have been associated with increased apoptosis of β-cells and a reduction in β-cell mass [34, 35]. Although our high-H population has a higher BMIp compared to the low-H group, these individuals are not overtly obese, with a median BMIp of 53.6. Boden et al. demonstrated a correlation between increased leptin secretion in non-obese patients and prolonged hyperinsulinemia [36], which is consistent with the findings in our high-H cohort. Additionally, elevated serum levels of PAI-1, an early inflammatory marker [37], were found to be influenced by BMIp as also reported by Koca et al. [38, 39]; while active amylin, a peptide secreted together with insulin by pancreatic β cells [40], correlated with HOMA-IR, confirming previous evidence [41, 42].

Unexpectedly, our data did not provide evidence of systemic inflammation. Specifically, TNF-α and IL-6, which have been reported to be elevated in individuals with obesity or T2D [43], were not found to be significantly increased in the high-H group. This lack of elevation may be attributed to the fact that only a small fraction of individuals in the high-H group are obese, with a median BMIp of 53.6, and none exhibit overt diabetes. Our data revealed increased engagement of islet-specific CD8 T cells in a subgroup of HLA-A2 + individuals with high-H, suggesting a possible link between insulin resistance and adaptive immune responses. Specifically, insulin resistance was associated with a memory profile as well as activation of GAD65-specific T cells. This phenomenon aligns with the findings of Viglietta V. et al., who observed an activated memory profile in GAD65-reactive T cells, a hallmark of T1D at onset [44]. Furthermore, we found no differences in the frequency of circulating effector GAD65-specific CD8 T cells, which were previously associated with C-peptide levels after diagnosis [45]. Even though the frequency and phenotype of circulating T cells does not necessarily mirror autoimmunity in the pancreas, together with the absence of β-cell stress, it suggests that a sustained immune assault on β-cells has not yet commenced.

In our study, insulin resistance is also correlated with an elevated frequency of insulin-specific CD8 T cells, independent of BMIp. However, these cells appear to maintain a naïve phenotype and exhibit heightened activation compared to the bulk CD8 T cell population, regardless of the state of insulin resistance. The presence of naïve β-cell specific CD8 T cells has been previously observed [46, 47]; however, it cannot be excluded the attribution of this phenotype to stem memory T cells (Tscm). Tscm have been previously observed in insulin-specific CD8 T cells [19, 48–51] and represent a subset of long-lived, self-renewing cells with a less differentiated profile similar to naïve cells and with an enhanced proliferative capacity comparable to that of CM T cells. Tscm could potentially act as a progenitor for the mediator cells responsible for β-cell destruction [19, 50]. Tscm share the naïve markers CD45RA and CCR7 but differ from naïve cells for the expression of CD95 [52]. It is worth noting that flow cytometry analysis in the current study did not encompass CD95, not allowing for the distinction between naïve and Tscm cells. Moreover, in light of recent findings highlighting GLP-1R as a T cell-negative costimulatory molecule, as well as the potential role of the immune checkpoint molecule PD-L1 in influencing T cell function, further studies are warranted to investigate how these receptors modulate islet-specific CD8 T cell responses [53, 54].

Our hypothesis postulates that islet-specific CD8 T cells emerge due to β-cell apoptosis within a critical timeframe when β-cells are strained by the compensatory mechanisms employed in response to insulin resistance. In conditions marked by β-cell damage and initial exposure to high glucose levels, β-cells express self-antigens [55, 56]. This, coupled with the observed hyper-expression of human leukocyte antigens of class-I (HLA-I) in the islets of autoantibody-positive relatives [57, 58], facilitates the recognition of β-cell antigen by specific CD8 T cells, triggering their activation and differentiation. It's important to note that we did not detect a significant increase in β-cell stress as indicated by the PI/C-peptide ratio, suggesting that it may still be at a subclinical stage. Islet-specific cells may play a role in causing damage to β-cells, and potentially can contribute to the systemic and tissue-specific chronic inflammation observed in insulin resistance [6].

Limitations include the small cohort of relatives studied in this project that could affect the lack of differences observed in the genetic predisposition. Nevertheless, it has to be acknowledged that our study did not specifically investigate the genetic predisposition associated with insulin resistance and T2D [59]. Therefore, we cannot definitively assert whether environmental factors, rather than T2D genetic predisposition, significantly contribute to insulin resistance in the context of T1D. Furthermore, due to the lack of a long-term follow-up, we were unable to investigate how these features affected the risk of developing T1D. Strengths include the multiple assessments and associations that provide a comprehensive overview of the effects on insulin resistance in individuals at risk for developing T1D.

Collectively, our findings suggest that insulin resistance in relatives of T1D patients leads to metabolic alterations for glucose level maintenance and distinct islet-specific cell frequency and phenotype. This metabolic and immunological feature may render β-cells more susceptible to autoimmune attacks, potentially explaining the increased susceptibility of insulin-resistant relatives to developing T1D. However, the precise mechanisms underlying these processes remain incompletely understood. The complex interplay between the immune system and metabolic factors underscores the need for further research to unravel these connections.

## Supplementary Information

Below is the link to the electronic supplementary material.

Supplementary file 1 (DOCX 526 KB)

## Data Availability

Original data generated and analysed during this study are available upon request to the corresponding author**.**
