# Supplementary material for: Metabolic, genetic and immunological features of relatives of type 1 diabetes patients with elevated insulin resistance
Source: J Endocrinol Invest. 2024 Dec 10;48(3):765–75. doi: 10.1007/s40618-024-02497-x (PMC11876269; doi:10.1007/s40618-024-02497-x)
Supplement: Supplementary file 1 — (DOCX 526 KB) [file 40618_2024_2497_MOESM1_ESM.docx]

**SUPPLEMENTARY MATERIAL**

**SUPPLEMENTARY TABLES**

| \| **Antibody** \| **Fluorophore** \| **ul/Sample** \| **Company** \| **Order no** \| **Peptide sequence** \| \| --- \| --- \| --- \| --- \| --- \| --- \| \| HLA-A2 \| PE \| 1 \| Biolegend \| 343306 \|  \| \| Horizon Fixable Viability Stain 575V \|  \| 1 \| BD \| 565694 \|  \| \| CD8 \| APC-H7 \| 0.2 \| BD \| 560179 \|  \| \| CD45 \| PERCPCY5 \| 0.25 \| Biolegend \| 304028 \|  \| \| CD69 \| BV650 \| 0.25 \| Biolegend \| 310934 \|  \| \| CD137 \| BUV737 \| 0.25 \| BD \| 741861 \|  \| \| LAG-3 \| PECF594 \| 0.5 \| BD \| 565719 \|  \| \| CD45RA \| PeVio770 \| 0.5 \| Miltenyi \| 130-097-577 \|  \| \| CD154 \| BV480 \| 0.5 \| BD \| 746337 \|  \| \| CD56 \| BUV563 \| 0.5 \| BD \| 612929 \|  \| \| CD4 \| BUV805 \| 0.5 \| BD \| 612887 \|  \| \| CD3 \| BUV395 \| 1 \| BD \| 564001 \|  \| \| CCR7 \| AF647 \| 1.5 \| BD \| 560816 \|  \| \| Insulin \| PE \| 5 \| Immudex \| WB03404 PE 150 \| HLAA*0201 / ALWGPDPAAA / PE \| \| GAD65 \| FITC \| 5 \| Immudex \| WB03405 FITC 150 \| HLAA*0201 / VMNILLQYVV / FITC \|   **Supplementary Table 1: Flow cytometry antibodies panel.** List of anti-human monoclonal antibodies and dextramers for anti-GAD65 and anti-insulin used in flow cytometry analysis; fluorophore, concentration, company, catalogue number and peptide sequence (for dextramers) are reported. |
| --- | --- | --- | --- | --- | --- | --- | --- | --- | --- | --- | --- | --- | --- | --- | --- | --- | --- | --- | --- | --- | --- | --- | --- | --- | --- | --- | --- | --- | --- | --- | --- | --- | --- | --- | --- | --- | --- | --- | --- | --- | --- | --- | --- | --- | --- | --- | --- | --- | --- | --- | --- | --- | --- | --- | --- | --- | --- | --- | --- | --- | --- | --- | --- | --- | --- | --- | --- | --- | --- | --- | --- | --- | --- | --- | --- | --- | --- | --- | --- | --- | --- | --- | --- | --- | --- | --- | --- | --- | --- | --- | --- | --- | --- | --- | --- | --- |

|  |  |  |  |  |  |
| --- | --- | --- | --- | --- | --- |
| **Inflammatory cytokines (pg/mL)** | **Low-H** | **High-H** | **p-value** | **Correlation with HOMA-IR** |  |
| Growth related oncogene-alpha (GROα) | 315.5 (164.0-575.5) | 211.0 (158.5-462.0) | 0.45 | r=-0.0034  p=0.98 |  |
| Interferon-alpha2 (IFNα2) | 11.0 (8.9-12.6) | 10.0 (9.0-13.0) | 0.96 | r=0.0296  p=0.83 |  |
| Interferon gamma (IFNγ) | 37.5 (30.0-57.8) | 40.0 (32.0-52.0) | 0.65 | r=0.2073  p=0.12 |  |
| Interleukin 1 beta (IL-1β) | 20.8 (17.0-26.0) | 21.0 (15.0-32.0) | 0.74 | r=0.0162  p=0.91 |  |
| Interleukin 1 receptor antagonist (IL-1RA) | 31.8 (21.6-45.3) | 31.5 (22.5-46.0) | 0.73 | r=0.0320  p=0.81 |  |
| Interleukin 13 (IL-13) | 27.5 (20.9-39.4) | 27.5 (22.0-38.5) | 0.93 | r=0.1222  p=0.37 |  |
| Interleukin 15 (IL-15) | 35.3 (27.0-41.1) | 32.5 (26.0-37.0) | 0.36 | r=-0.0224  p=0.87 |  |
| Interleukin 17-A (IL-17A) | 15.0 (13.0-17.3) | 16.0 (13.5-22.5) | 0.37 | r=0.0694  p=0.61 |  |
| Interleukin 6 (IL-6) | 26.8 (23.9-35.1) | 30.0 (24.5-41.0) | 0.30 | r=0.2081  p=0.12 |  |
| Interleukin 8 (IL-8) | 135.3 (97.1-197.8) | 119.5 (73.5-176.0) | 0.19 | r=-0.0437  p=0.75 |  |
| Interleukin 10 (IL-10) | 20.0 (15.1-28.0) | 19.0 (16.0-27.0) | 0.63 | r=0.0589  p=0.66 |  |
| Interferon-gamma inducible protein 10kDa (IP-10) | 687.3 (412.8-933.9) | 507.0 (361.0-864.0) | 0.47 | r=-0.0054  p=0.97 |  |
| monocyte chemoattractant protein 1 (MCP1) | 2120.0 (1628.0-3608.0) | 2182.0 (1443.0-3216.0) | 0.79 | r=0.0465  p=0.73 |  |
| Macrophage inflammatory protein-1 alpha (MIP-1α) | 18.3 (15.0-26.1) | 18.0 (13.5-28.0) | 0.65 | r=-0.0857  p=0.53 |  |
| Soluble CD40 ligand (sCD40L) | 2630.0 (1854.0-4875.0) | 2478.0 (1565.0-4889.0) | 0.76 | r=0.0178  p=0.90 |  |
| Tumor necrosis factor alpha (TNFa) | 33.8 (28.0-44.6) | 39.0 (27.0-52.0) | 0.45 | r=0.1984  p=0.14 |  |
|  | | | | | |
| **Supplementary Table 2: Inflammatory Cytokines in high-H vs low-H.** Serum levels of inflammatory cytokines for both low-H and high-H groups are shown. Data is expressed as the median and interquartile range. Statistical analysis was performed using the Mann-Whitney test. Correlations with HOMA-IR were assessed using the Spearman's rank-order correlation analysis. *HOMA-IR - Homeostatic Model Assessment* of Insulin Resistance*.* | | | | | |

|  | **Low-H**  **(n=8)** | **High-H**  **(n=12)** | **p-value** |
| --- | --- | --- | --- |
| Sex, F (%) | 4 (50.0) | 7 (58.3) | p>0.99 |
| Age (yrs)  <18 yrs  >18 yrs | 16.7 (8.9-36.2)  10.1 (5.4-13.9)  n=4  34.3 (21.9-44.5)  n=4 | 19.6 (14.4-37.6)  14.0 (7.8-16.4)  n=5  20.1 (20.0-46.0)  n=7 | p=0.62  p=0.41  p=0.89 |
| BMIp | 44.7 (3.4-58.1)  n=7 | 84.5 (31.7-94.4)  n=12 | P=0.10 |
| HOMA-IR | 0.6 (0.3-0.7)  n=8 | 2.3 (1.9-2.9)  n=12 | p<0.0001 |
| Matsuda | 13.3 (11.1-22.6)  n=8 | 4.4 (3.4-5.3)  n=12 | p<0.0001 |
| IGI | 28.8 (23.8-47.2)  n=8 | 129.5 (53.8-160.9)  n=12 | p=0.006 |
| C-peptide (AUC) | 414.7 (376.2-591.7)  n=8 | 850.6 (669.4-1056.0)  n=12 | p=0.0011 |
| HbA1c % | 5.1 (4.9-5.4)  n=7 | 5.0 (4.8-5.5)  n=12 | p=0.76 |
| Dysglycemia, n (%) | 5 (62.5) | 5 (41.7) | p=0.65 |
| N Aab  0, n (%)  1, n (%)  2, n (%)  3, n (%)  4, n (%)  5, n (%)  Unknown, n (%) | 3 (37.5)  2 (25)  1 (12.5)  1 (12.5)  1 (12.5) | 1 (8.3)  5 (41.7)  3 (25.0)  2 (16.7)  1 (8.3) |  |
| Type of Aab  GAD65, n (%)  ZnT8, n (%)  IA2, n (%)  mIAA, n (%)  ICA, n (%) | 4 (50.0)  1 (12.5)  1 (12.5)  2 (25) | 10 (83.3)  4 (33.3)  3 (25.0)  1 (8.3)  3 (25) |  |

**Supplementary Table 3: Clinical characteristics of low-H and high-H relatives of T1D selected for flow cytometry analysis.** Data are expressed as the median and interquartile range for continuous variables (age, BMIp, HOMA-IR, Matsuda, IGI, C-peptide (AUC), HbA1c), and as absolute numbers and percentages for nominal variables (sex, dysglycemia, number and type of autoantibodies positive patients). P-values were calculated using the Mann-Whitney test for continuous variables and Fisher’s exact test for nominal variables. *BMIp - Body Mass Index Percentile; HOMA-IR - Homeostatic Model Assessment* of Insulin Resistance*; IGI - Insulinogenic Index; AUC - Area Under the Curve;* *HbA1c - Glycated Hemoglobin.*

|  | **Variables** | **Unadjusted p-value** | **BMIp-adjusted p-value** |
| --- | --- | --- | --- |
| Metabolic variables | BMIp | 0.004 | NA |
|  | HOMA-IR | <0.0001 | <0.0001 |
|  | Matsuda | <0.0001 | <0.0001 |
|  | IGI | 0.001 | 0.01 |
|  | C-peptide (AUC) | 0.0003 | 0.01 |
|  | HbA1c % | 0.18 | 0.10 |
|  | Glucose t=0 min | 0.006 | 0.03 |
|  | Glucose t=30 min | 0.77 | 0.78 |
|  | Glucose t=60 min | 0.67 | 0.91 |
|  | Glucose t=90 min | 0.46 | 0.41 |
|  | Glucose t=120 min | 0.15 | 0.08 |
|  | Insulin t=0 min | <0.0001 | <0.0001 |
|  | Insulin t=30 min | 0.0002 | 0.006 |
|  | Insulin t=60 min | <0.0001 | 0.003 |
|  | Insulin t=90 min | 0.0006 | 0.005 |
|  | Insulin t=120 min | <0.0001 | <0.0001 |
|  | C-peptide t=0 min | <0.0001 | <0.0001 |
|  | C-peptide t=30 min | 0.0003 | 0.02 |
|  | C-peptide t=60 min | 0.001 | 0.04 |
|  | C-peptide t=90 min | 0.005 | 0.06 |
|  | C-peptide t=120 min | 0.001 | 0.01 |
|  | PI/C-peptide | 0.66 | 0.15 |
|  | Leptin | <0.0001 | <0.0001 |
|  | Amylin active | 0.71 | 0.89 |
|  | PAI-1 | 0.01 | 0.11 |
| Genetic variable | Genetic risk score 1 (GRS1) | 0.23 | 0.30 |
| Immunological variables | GAD65-specific CD8 T cells | 0.45 | 0.23 |
|  | CM GAD65-specifc CD8 T cells | 0.06 | 0.05 |
|  | CD69+ GAD65-specific T cells | 0.11 | 0.05 |
|  | CD154+ GAD65-specific T cells | 0.11 | 0.12 |
|  | CD137+ GAD65-specific T cells | 0.05 | 0.17 |
|  | LAG3+ GAD65-specific T cells | 0.03 | 0.02 |
|  | Insulin-specific CD8 T cells | 0.02 | 0.02 |
|  | CD69+ insulin-specific CD8 T cells | 0.67 | 0.84 |
|  | CD154+ insulin-specific CD8 T cells | 0.38 | 0.77 |
|  | CD137+ insulin-specific CD8 T cells | 0.85 | 0.43 |
|  | LAG3+ insulin-specific CD8 T cells | 0.37 | 0.67 |

**Supplementary Table 4: Influence of BMIp adjustment on metabolic, genetic and immunologic characteristics.** P-values were calculated using a linear regression model with and without BMIp as a covariate. Statistical unadjusted p-value and adjusted p-value for each variable are reported**.** *BMIp - Body Mass Index Percentile; HOMA-IR - Homeostatic Model Assessment of Insulin Resistance; IGI - Insulinogenic Index; AUC - Area Under the Curve; HbA1c - Glycated Hemoglobin; PI - Proinsulin; PAI-1 - Plasminogen Activator Inhibitor-1; GRS1 - T1D Genetic Risk Score 1; CM - Central Memory.*

**SUPPLEMENTARY FIGURES**

| 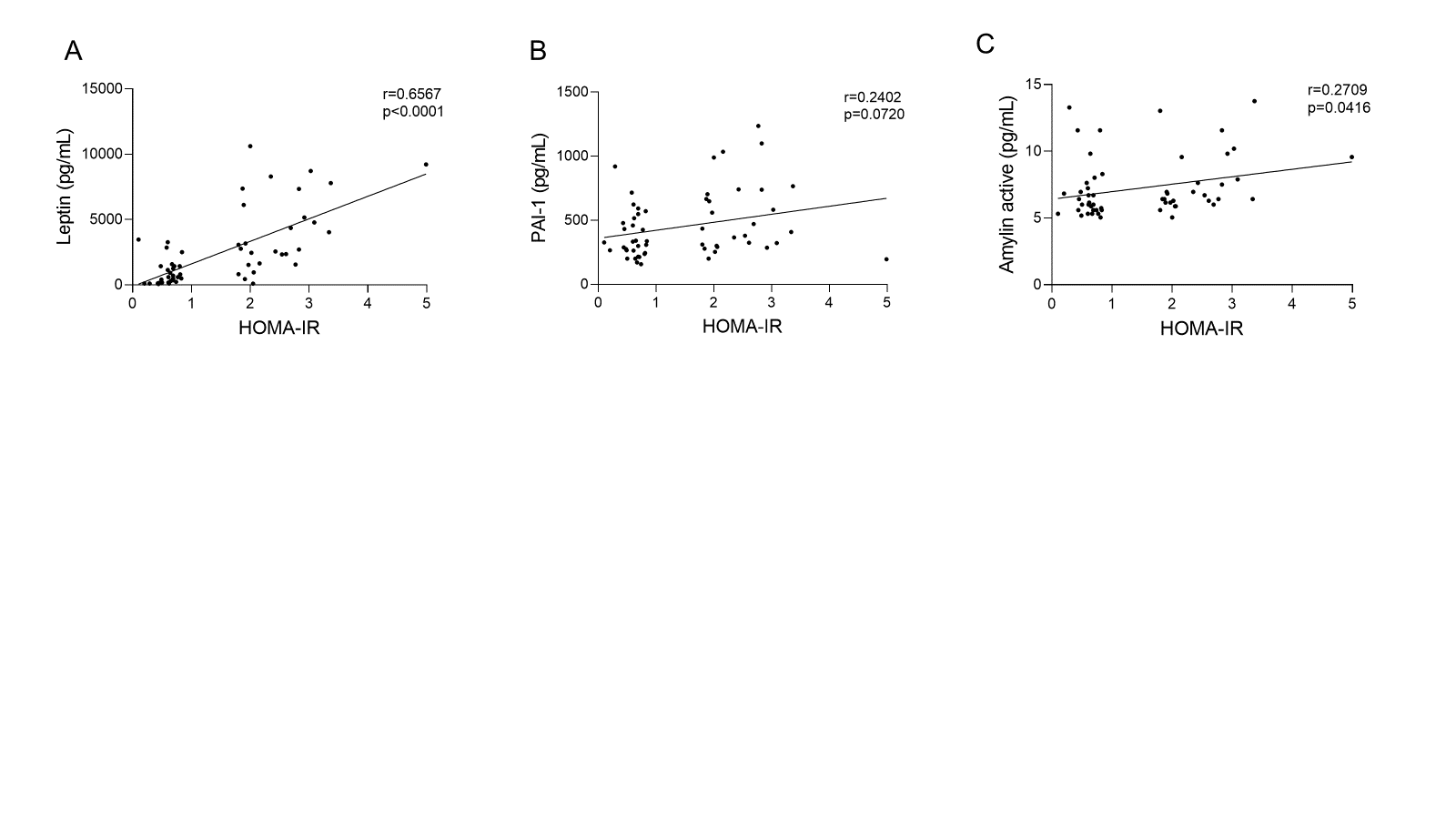 |
| --- |
| **Supplementary Figure 1: Correlations of leptin, PAI-1 and amylin active with HOMA-IR.** Correlation between leptin (A), PAI-1 (B) and amylin active (C) and HOMA-IR. Correlations were assessed using Spearman's rank-order correlation analysis. *HOMA-IR - Homeostatic Model Assessment* of Insulin Resistance*; PAI-1 - Plasminogen Activator Inhibitor-1.* |


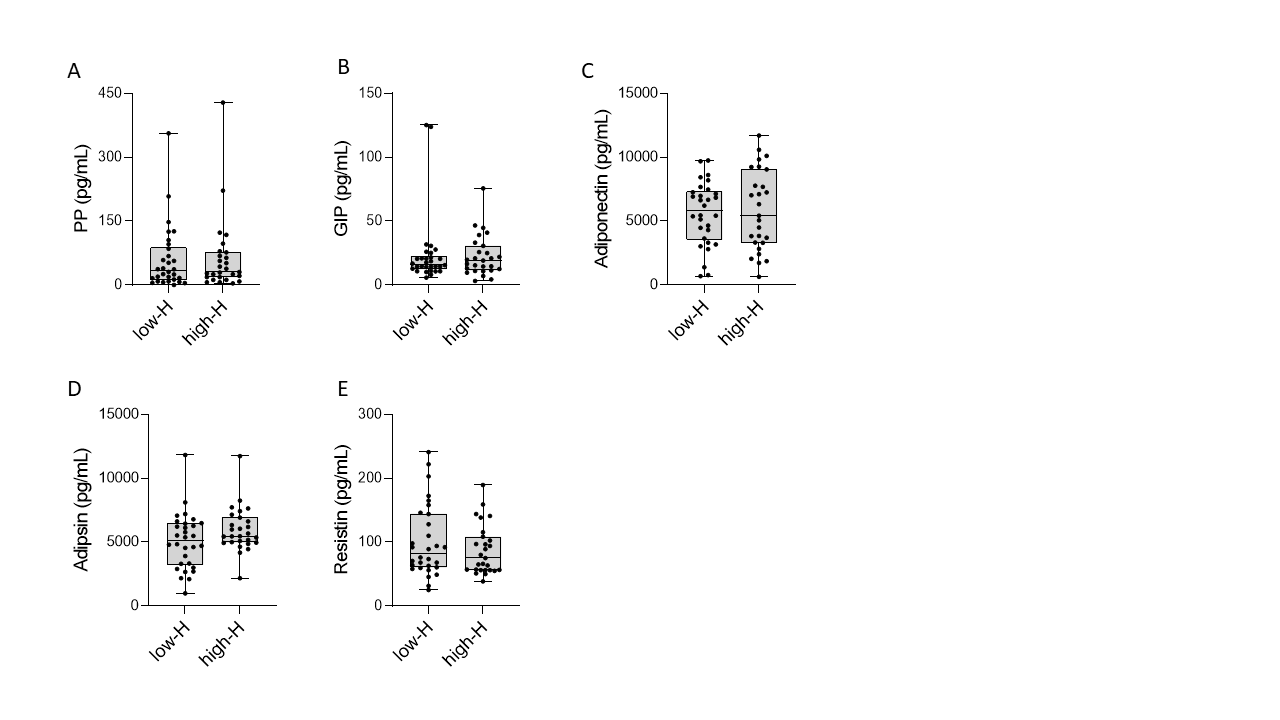


| **Supplementary Figure 2: Serum metabolic hormones in high-H vs low-H**. Comparison between low-H and high-H for PP (A), GIP (B), adiponectin (C), adipsin (D), resistin (E). All graphs are presented as the median and interquartile range (IQR). Statistical analysis was performed using the Mann-Whitney test. *PP - Pancreatic Polypeptide; GIP - Gastric Inhibitory Polypeptide.* |
| --- |

|  |
| --- |
| ****  **Supplementary Figure 3: Correlations of GRS1 with HOMA-IR**. Correlation between GRS1 and HOMA-IR. Correlations were assessed using the Spearman's rank-order correlation analysis. *GRS1- T1D Genetic risk score 1; HOMA-IR - Homeostatic Model Assessment* of Insulin Resistance*.* |

| **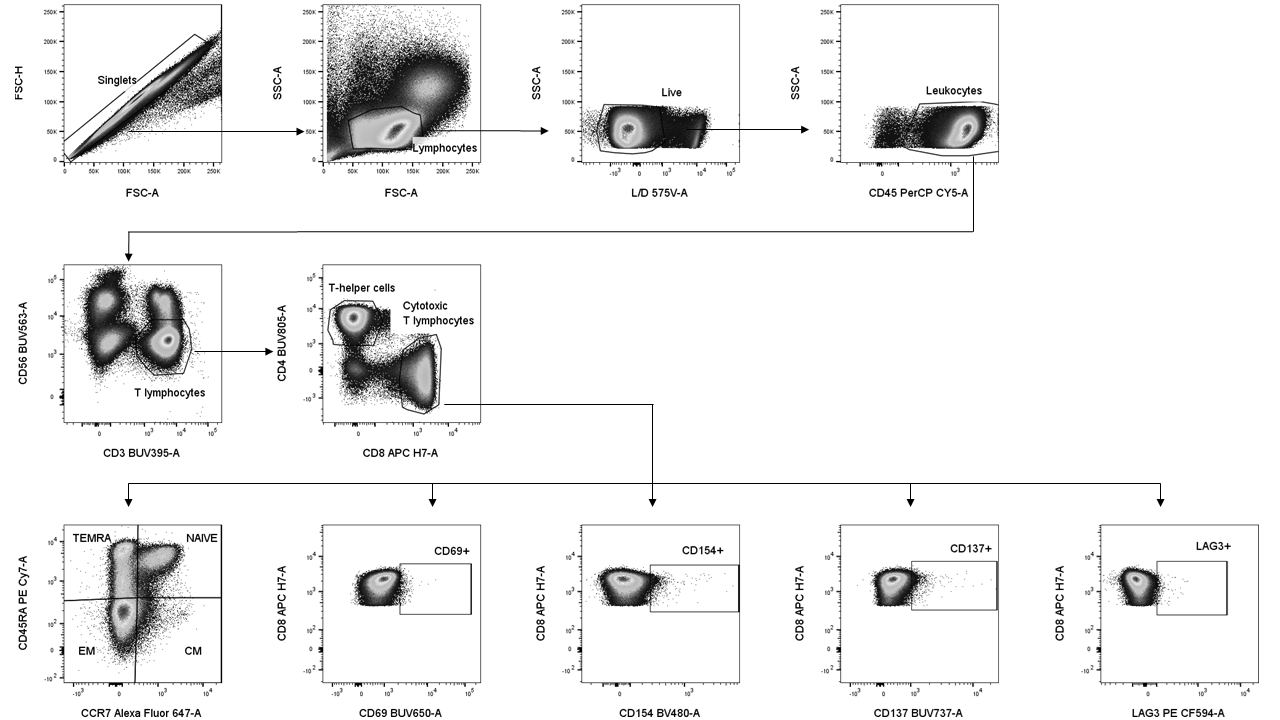** |
| --- |
| **Supplementary Figure 4: Gating strategy of flow cytometry analysis.** Singlets and lymphocytes were selected based on physical parameters. Among lymphocyte, live cells (L/D^-^), total leukocytes (CD45^+^), T lymphocytes (CD3^+^/CD56^-^), T-helper cells (CD4^+^/CD8^-^), and cytotoxic T lymphocytes (CD8^+^/CD4^-^) were gated. Among cytotoxic T lymphocytes, naïve (CD45RA^+^/CCR7^+^), central memory (CM) (CD45RA^-^/CCR7^+^), effector memory (EM) (CD45^-^/CCR7^-^), and terminally differentiated effector memory T cells (TEMRA) (CD45RA^+^/CCR7^-^) were identified. Additionally, cell activation was evaluated using CD69, CD154, CD137, and LAG3 markers. |

| **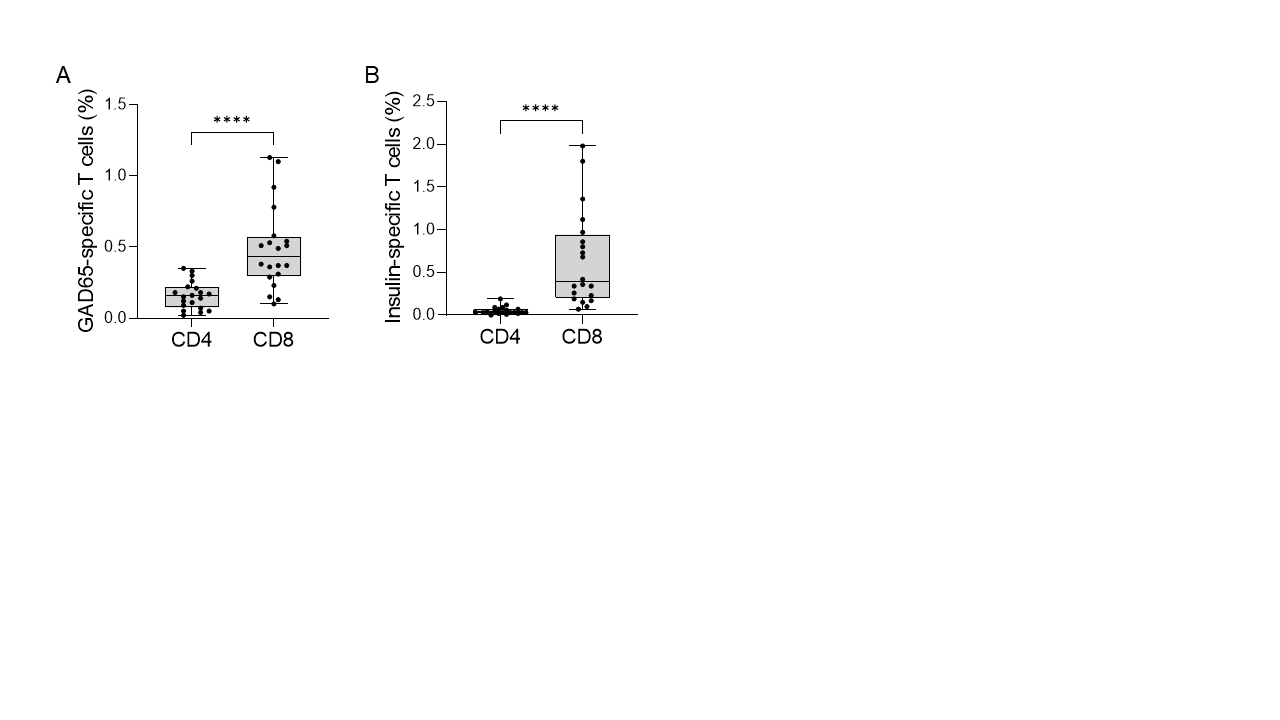** |
| --- |
| **Supplementary Figure 5: Negative controls for islet-specific CD8 T cells**. The frequency of GAD65-specific (A) and insulin-specific cells (B) gated on CD8 T cells were compared with CD4 T cells, which served as negative control. The graphs display the median values along with the interquartile range (IQR). Statistical analysis was performed using the Mann-Whitney test. |

|  |
| --- |
| **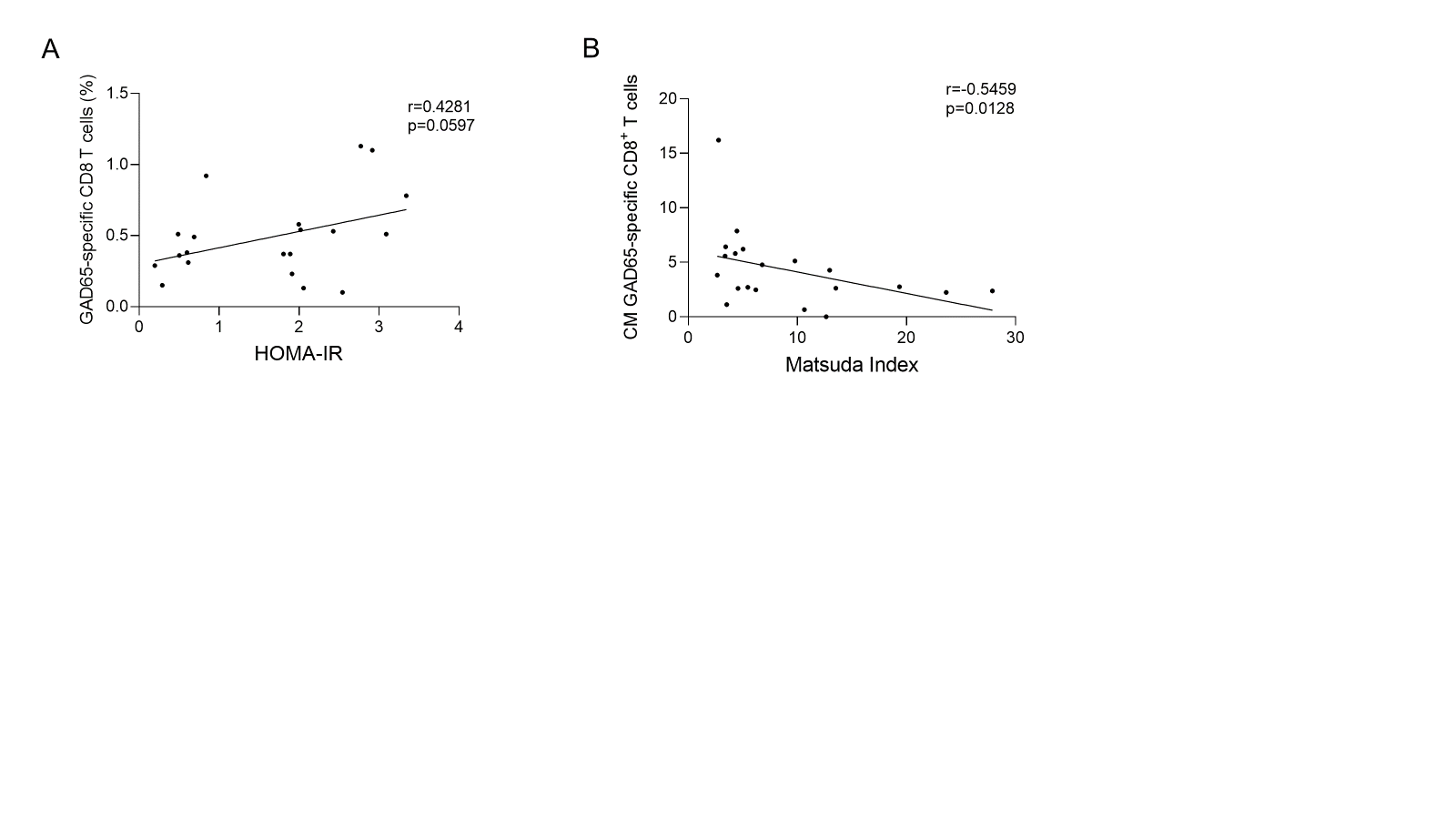**  **Supplementary Figure 6: Correlations of GAD65-specific CD8 T cells with HOMA-IR and Matsuda index.** Correlation between GAD65-specific CD8 T cells and HOMA-IR (A). Correlation between CM GAD65-specific CD8 T cells and Matsuda index (B). Spearman’s rank-order correlation analysis was used. *HOMA-IR - Homeostatic Model Assessment* of Insulin Resistance*; CM - Central Memory.* |
|  |

**
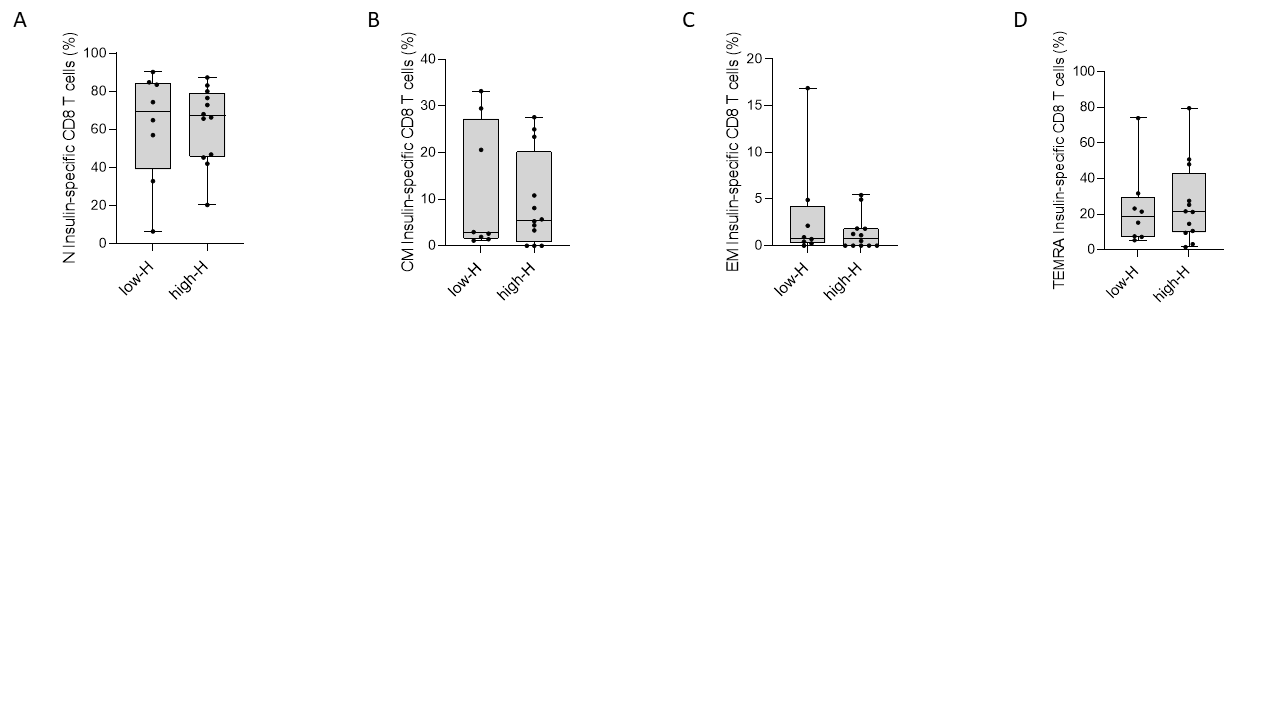
**

| **Supplementary Figure 7: Phenotype of insulin-specific CD8 T cells in high-H vs low-H.**  Comparison of N insulin-specific CD8 T cell frequency between low-H and high-H groups (A). Comparison of CM insulin-specific CD8 T cells frequency between low-H and high-H groups (B). Comparison of EM insulin-specific CD8 T cell frequency between low-H and high-H groups (C). Comparison of TEMRA insulin-specific CD8 T cells frequency between low-H and high-H groups (D). All graphs are presented as the median and interquartile range (IQR). Statistical significance (p-value) was determined using the Mann-Whitney test. * p<0.05, **p<0.01, *** p<0.001, **** p<0.0001. *N - Naïve*; *CM - Central Memory; EM - Effector Memory; TEMRA - Terminally Differentiated Effector Memory Cells.* |
| --- |
|   **Supplementary Figure 8: Correlation of insulin-specific CD8 T cells with Matsuda index**. Correlation between insulin-specific CD8 T cells and Matsuda index. The correlations were evaluated using the Spearman’s rank-order correlation analysis. |

|  |
| --- |
| **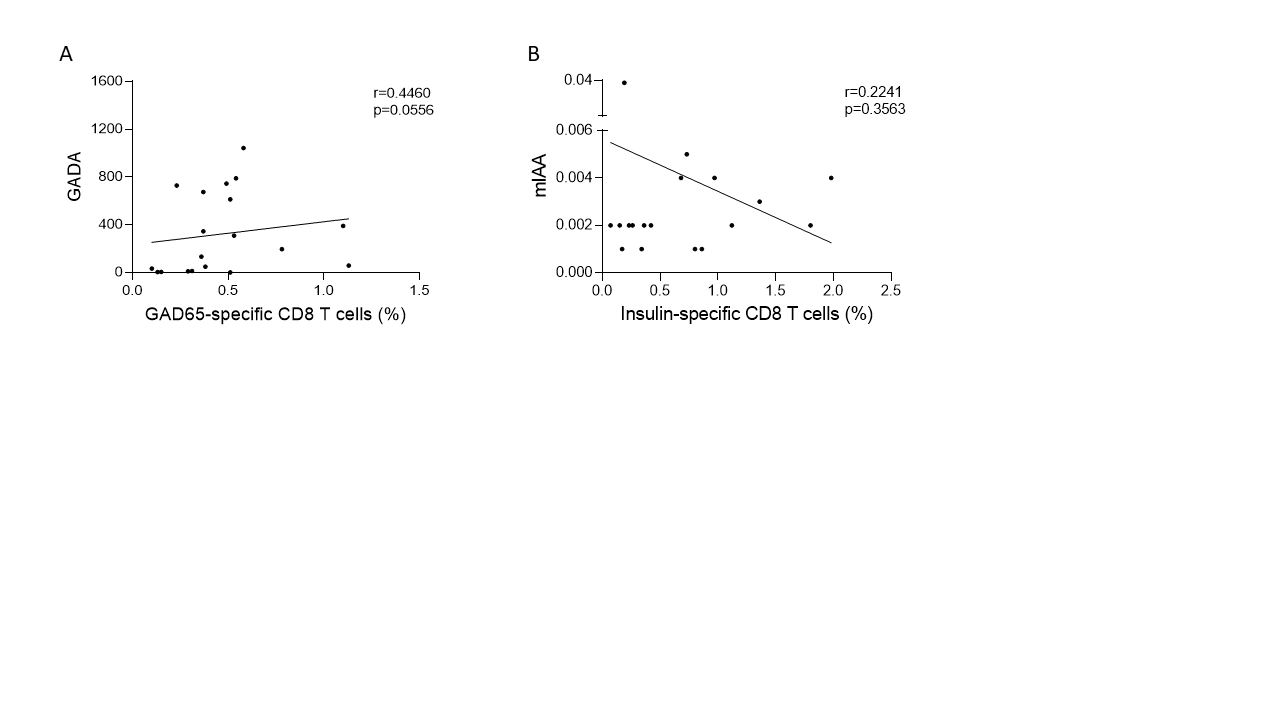**  **Supplementary Figure 9: Correlations of GAD65- and insulin-specific CD8 T cells with anti-GAD65 and anti-insulin autoantibodies.** Correlation between GADA levels and GAD65-specific CD8 T cells (A). Correlation between mIAA levels and insulin-specific CD8 T cells (B). Correlation was performed with Spearman’s rank-order correlation analysis. *GADA - Anti-Glutamic Acid Decarboxylase Antibody; mIAA - Anti-Insulin Antibody.* |
